# Accounting for uncertainty in training data to improve machine learning performance in predicting new disease activity in early multiple sclerosis

**DOI:** 10.3389/fneur.2023.1165267

**Published:** 2023-05-26

**Authors:** Maryam Tayyab, Luanne M. Metz, David K.B. Li, Shannon Kolind, Robert Carruthers, Anthony Traboulsee, Roger C. Tam

**Affiliations:** ^1^School of Biomedical Engineering, University of British Columbia, Vancouver, BC, Canada; ^2^Cumming School of Medicine, University of Calgary, Calgary, AB, Canada; ^3^Department of Radiology, Faculty of Medicine, University of British Columbia, Vancouver, BC, Canada; ^4^Division of Neurology, Department of Medicine, University of British Columbia, Vancouver, BC, Canada

**Keywords:** multiple sclerosis, machine learning, MRI, label uncertainty, clinical prediction, missing labels, noisy labels

## Abstract

**Introduction:**

Machine learning (ML) has great potential for using health data to predict clinical outcomes in individual patients. Missing data are a common challenge in training ML algorithms, such as when subjects withdraw from a clinical study, leaving some samples with missing outcome labels. In this study, we have compared three ML models to determine whether accounting for label uncertainty can improve a model’s predictions.

**Methods:**

We used a dataset from a completed phase-III clinical trial that evaluated the efficacy of minocycline for delaying the conversion from clinically isolated syndrome to multiple sclerosis (MS), using the McDonald 2005 diagnostic criteria. There were a total of 142 participants, and at the 2-year follow-up 81 had converted to MS, 29 remained stable, and 32 had uncertain outcomes. In a stratified 7-fold cross-validation, we trained three random forest (RF) ML models using MRI volumetric features and clinical variables to predict the conversion outcome, which represented new disease activity within 2 years of a first clinical demyelinating event. One RF was trained using subjects with the uncertain labels excluded (RF_exclude_), another RF was trained using the entire dataset but with assumed labels for the uncertain group (RF_naive_), and a third, a probabilistic RF (PRF, a type of RF that can model label uncertainty) was trained on the entire dataset, with probabilistic labels assigned to the uncertain group.

**Results:**

Probabilistic random forest outperformed both the RF models with the highest AUC (0.76, compared to 0.69 for RF_exclude_ and 0.71 for RF_naive_) and F1-score (86.6% compared to 82.6% for RF_exclude_ and 76.8% for RF_naive_).

**Conclusion:**

Machine learning algorithms capable of modeling label uncertainty can improve predictive performance in datasets in which a substantial number of subjects have unknown outcomes.

## 1. Introduction

Multiple sclerosis (MS) is a chronic autoimmune disease of the central nervous system (CNS) characterized by neuroinflammation, demyelination, and neurodegeneration. While there is no cure for MS, evidence has shown that early diagnosis and early treatment can improve long-term prognosis in people that are at high risk of progressing in their disease (1). MS disease course is highly heterogeneous, especially in the early stages, and it is difficult to identify individuals who stand to benefit from more early management. In individuals presenting with the early signs and symptoms suggestive of MS, a combination of clinical signs and symptoms and imaging-based indicators, formalized in the form of the McDonald criteria (2, 3), are used to establish a diagnosis. Predicting whether an individual in the early phase of MS will meet certain progression and conversion criteria within a given time remains challenging.

Damage to the myelin sheath is the most obvious manifestation of MS, particularly in the form of white matter lesions as seen on magnetic resonance images (MRIs). While WM lesion volume, also known as the burden of disease (BOD), is a commonly used imaging biomarker to evaluate disease progression and treatment response in clinical studies, it does not capture MS pathology comprehensively and on its own is a limited predictor of future disease activity. Demyelination and morphological changes in deep gray matter (DGM) are recognized as consistent and clinically relevant features in all MS phenotypes (4–6) and several studies have found associations between DGM volume loss and the severity of physical and cognitive impairment (4–8). Volume loss in the thalamus has been linked with clinical worsening in individuals with early symptoms of MS (9) and has been observed in both clinically isolated syndrome (CIS) (10) and radiologically isolated syndrome (RIS) (11) populations compared to healthy controls. However, further investigations are needed to determine the value of DGM atrophy as a predictor of future disease development and to optimize the analysis of DGM volume changes to inform clinical decision-making (6).

Within the scope of MS, machine learning (ML) methods have been proposed mainly for early detection, differentiation of MS phenotypes from each other and healthy controls (12, 13), and prediction of disease and disability progression (14–16). Several studies have used ML models to predict the conversion of CIS to confirmed MS, based on clinical, demographic, and radiologic features with varying degrees of success (17–20). However, no prior study has focused specifically on the value of DGM volumes in the prediction of new disease activity in the very early stages of MS. In this study, we apply ML to DGM volumes for clinical prediction while also including BOD as a variable.

The lack of sufficiently large labeled clinical datasets has been a major limiting factor in training machine learning models for clinical prediction (21). Due to the considerable costs associated with curating and labeling imaging datasets, there is a paucity of publicly available data, and even most proprietary datasets are small in the context of ML applications. For most clinical datasets, missing or uncertain labels are a common occurrence (21). Literature on ML training has shown that noisy labels can adversely affect the model performance especially if the sample size and/or the number of variables is small (22–26).

Several ML approaches have been proposed to represent and integrate the degree of uncertainty in the class labels into model training. For example, support vector machines (SVMs) have been formulated with uncertainty-weighted training schemes (27–29). Others have proposed methods based on the random forest (RF), such as noise-tolerant random forest (RF) (30), noise-tolerant loss functions (31), and probabilistic random forest (PRF) (32). PRF handles noisy, uncertain, and missing labels and input features by modeling them as distribution functions instead of deterministic values (32). The algorithm was originally developed for dealing with missing and noisy data in astronomical datasets and has since been verified on bioactivity data with promising results (33), but has not been tested for predicting clinical outcomes.

In this paper, we compared three approaches for handling uncertain or missing class labels. Using a dataset with 142 patients, with 32 having uncertain labels, we compared an RF model trained only on the subset of confirmed class labels and excluding the uncertain samples (RF_exclude_), an RF model trained on the entire dataset treating the uncertain class labels as confirmed labels (RF_naive_), and a PRF model trained using the entire dataset but with uncertainty estimates incorporated in the model training. PRF was chosen based on the status of the RF as a leading ML method for tabular data (34), the relative simplicity of training, and promising performance in other data domains.

The models were trained to predict the development of new disease activity in MS within 2 years of the first clinical demyelinating event using clinical, demographic, and imaging-based variables. The goal was to determine whether excluding, ignoring, or modeling the uncertainty would result in the highest-performing model.

## 2. Methods

### 2.1. Dataset and study participants

The dataset for our experiment consisted of MRIs and clinical and demographic data from a completed multi-center, phase-III clinical trial conducted to determine the efficacy of minocycline for delaying conversion from CIS to clinically definitive MS diagnosed, using the McDonald 2005 criteria (2) within 6 months (primary trial endpoint) and 2 years (secondary trial endpoint) after randomization. The study population consisted of 142 participants between the ages of 18 and 60 years who had been diagnosed with CIS after experiencing a first demyelinating event and were recruited from 12 MS clinics across Canada between 2009 and 2013. The detailed study design and participant inclusion and exclusion criteria can be found in (35). The baseline characteristics of the participants are summarized in Table 1. By the end of 24 months, 81 (57.04%) participants had converted to MS as per the McDonald 2005 criteria, 26 (18.57%) participants dropped out of the trial due to various reasons (35) before meeting the secondary trial endpoint, and 35 (24.3%) remained stable. Of these 35 non-converters, six were recruited toward the end of the trial and were followed only for a year, thus their status at 2-year post-enrollment in the trial was unknown. Thus, in our dataset of 142 subjects, we had 32 subjects that had an unknown status at 24 months.

**Table 1:** Dataset characteristics.

Characteristics	Values
Total number of participants	142
Participants assigned to minocycline	72 (51.42%)
Participants assigned to placebo	70 (48.57%)
Mean age at CIS^a^ onset [years (SD)]	35.9 (9.2)
Number of females [*n* (%)]	97 (69%)
Number of participants from white race [*n* (%)]	120 (86%)
Median EDSS^b^ (range)	1.5 (0–4.5)
Median T2w lesion volume [mm^3^ (1st and 3rd Quartile)]	1675.15 (550.475, 3796.8)
Median CIS^a^ duration [days (range)]	83.5 (21–190)

^a^Clinically isolated syndrome.

^b^Expanded disability status scale.

For our experiment, an individual was considered to have developed new disease activity if they met the clinical trial’s primary or secondary endpoints. The primary endpoint of the study was the conversion to MS based on the McDonald 2005 criteria, 6 months after randomization. Secondary endpoints included conversion to MS within 24 months and several MRI outcomes, including changes in lesion volume on T2w MRI, the total number of enhancing lesions on T1w MRI, and the total number of unique new or newly enlarging lesions on both T1w and T2w MRIs at both 6 and 24 months. Even though the McDonald criteria used in the original trial (35) has since been revised several times to enable earlier diagnosis, most recently in 2017 (36), the 2005 version is still a valid indicator of new disease activity. These criteria define clinical, imaging, and biological markers of MS pathology, so a person newly meeting these criteria in a given timeframe can be considered as having had new disease activity, even if they had already been previously diagnosed with MS.

### 2.2. MRI acquisition and preprocessing

We utilized three MRI sequences, including proton density-weighted (PDw), T2-weighted (T2w), and T1-weighted (T1w) scans that were acquired through a standardized protocol followed across all 12 sites. The studies were performed on scanners from GE, Siemens, and Philips operating at field strengths ranging from 1.5 to 3.0 T. The PDw scans had a TE range of 8–20 ms and TR range of 2,000–3,400 ms, T2w scans had a TE range of 78–116 ms and TR range of 2,800–8,000 ms, and T1w images were obtained using an IR-prepped gradient echo sequence with a TR range of 5–13 ms, TE range of 2–4 ms, and TI range of 450–800 ms. The T1-weighted scans had an image size of 256 × 256 × 160 and an isotropic voxel size of 1.00 mm × 1.00 mm × 1.00 mm, while the PDw and T2w scans had an image size of 256 × 256 × 60 and a voxel size of 0.937 mm × 0.937 mm × 3.000 mm.

The T1w MRIs were processed to minimize the effect of field inhomogeneity and skull-stripped using advanced normalization tools (ANTs) (37). Lesion filling was performed on the skull-stripped brains before spatial normalization to a standard template from the OASIS dataset (38). The spatial normalization was performed using ANTs and consisted of rigid, affine, and deformable registration. The WM lesion masks used for lesion filling were generated with the PDw/T2w scans using a semi-automated lesion segmentation method (39) where seed placement was done by an expert rater. The spatially normalized T1w images were intensity normalized using the FMRIB software library (FSL) (40). ANTs multi-atlas segmentation with label fusion pipeline was used for performing segmentation of the DGM nuclei resulting in individual 3D segmentations of the left and right thalami, putamina, caudate nuclei, and globus pallidi. The volume for each DGM nucleus was calculated (in mm^3^) using ANTs.

### 2.3. Input variables and prediction outcome

We started with 19 baseline variables collected from the trial participants, including 10 MRI volumetric measurements, six variables related to the type and anatomical location (s) of CIS onset, and three other variables (biological sex, EDSS, and treatment arm). A summary of these variables is presented in Table 2. The treatment group (i.e., minocycline or placebo) was included as an input variable because the original study showed that minocycline delayed the risk of conversion to MS by 18% within 6 months, although these results were not observed at 24 months. As described earlier, 26 participants had dropped out before meeting an endpoint, which may have affected the ability to detect a treatment effect at 24 months. The volumetric MRI measures included the volumes of individual DGM nuclei (eight in total), whole brain volume measured as brain parenchymal fraction (BPF), and WM lesion volume. Previous studies have shown that feature selection by excluding redundant or collinear features helps prevent the model from learning spurious correlations and hence results in better generalizability (20, 41, 42). In our previous work with the current data (43), we compared several automatic and manual methods for selecting input features and found that most methods ranked lesion volume, treatment arm, and subsets of the DGM volumes as the most important features. In the end, we found that including all eight DGM volumes, lesion volume, and treatment group resulted in the best model performance using an RF. Therefore, we used the same 10 features in this work.

**Table 2:** User-defined MRI and clinical measurements at baseline.

Parameter name	Measures
BOD	Burden of disease [T2w lesion volume (mm^3^)]
BPF	BPF (ratio of brain parenchymal volume to total intracranial volume)
Sex	Sex (Female = 1, Male = 0)
CIS type	CIS is monofocal at onset (=0) CIS is multifocal at onset (=1)
Cerebrum	Initial CIS event at cerebrum^*^ (Yes = 1, No = 0)
Optic nerve	Initial CIS event at optic nerve^*^ (Yes = 1, No = 0)
Cerebellum	Initial CIS event at cerebellum^*^ (Yes = 1, No = 0)
Brainstem	Initial CIS event at brain stem^*^ (Yes = 1, No = 0)
Spinal cord	Initial CIS event at spinal cord^*^ (Yes = 1, No = 0)
EDSS	Extended disability status scale
Mino	Indicates whether a participant was assigned to the treatment group (=1) or placebo group (=0)
R_caudate, L_caudate	Volumes of the right and left caudate nuclei (mm^3^)
R_thalamus, L_thalamus	Volumes of the right and left thalami (mm^3^)
R_putamen, L_putamen	Volumes of the right and left putamina (mm^3^)
R_palllidum, L_pallidum	Volumes of the right and left globus palidi (mm^3^)

^*^Anatomic location deemed to be the site of the first CIS attack, as determined by the physician.

The outcome of interest was whether new disease activity was observed within 24 months of enrollment in the study. Class labels for binary classification were assigned based on a participant’s clinical status at the end of 24 months in the trial (i.e., stable vs. developed new disease activity). This led to 81 subjects being labeled as having new disease activity while 29 were labeled as stable. Subjects who dropped out early or were only followed up for a year while still stable were assigned the *post hoc* label of stable as in previous ML studies (44), which applied to a total of 32 subjects. Although *post hoc* labels were assigned after deliberation among the researchers and clinicians involved in the studies, they were not confirmed and therefore had a level of uncertainty.

### 2.4. Machine learning models and training

We compared three approaches for dealing with the uncertain *post hoc* assigned labels in our dataset. The first approach excluded the uncertain data points, and a classic RF was trained on the remaining subset. The second approach trained a classic RF on the entire dataset and assumed the *post hoc* labels were correct. The third approach trained a PRF on the entire dataset, assigning 100% probability to the confirmed labels and 50% to the uncertain labels.

Random forest is a widely popular machine learning algorithm and a classic example of an ensemble classifier that is built from multiple unique decision trees (DTs) (45) that are individually constructed and trained using randomly selected subsets of input features and bootstrapped samples from the training dataset. During training, the algorithm learns to split the data into subsequent nodes in a way that reduces the class heterogeneity in the resulting child nodes. The trees are grown until a prespecified depth is reached or if the class heterogeneity can no longer be reduced. The node-splitting criterion, based on feature thresholds, forms the basis of the decision rules learned by a DT. The final prediction is provided by aggregating class predictions from the individual DTs through either a majority or average voting scheme.

### 2.5. Probabilistic random forest

The PRF is a modification of the RF built specifically to account for uncertainties in the input features and class labels in a dataset. While the input to an RF is of the form $\left(x_{i},y_{i}\right)$ , where $x_{i}$ is a feature vector and $y_{i}$ is its associated label, a PRF takes data points of a form $\left(x_{i},y_{i},\Delta x_{i},\Delta y_{i}\right)$ where $\Delta x_{i}$ is the uncertainty associated with $x_{i}$ and $\Delta y_{i}$ is the uncertainty associated with $y_{i}$ . In the absence of $\Delta x_{i}$ and $\Delta y_{i}$ , the PRF converges to an RF.

A PRF accounts for the uncertainties in the input features and class labels by modeling them as probability functions instead of deterministic values. The input features become probability distribution functions where the expected value of the distribution is the value provided for the feature and the variance of the distribution is the square of the uncertainty associated with that feature. The class labels on the other hand become probability mass functions and each class instance is treated as a class label with a probability determined by $\Delta y_{i}$ .

The details of the PRF algorithm are given in (32), but briefly, a PRF bags features by sampling them from the given distributions, propagates the features at each node to both child nodes with the associated class probabilities and performs splitting using modified Gini impurity function that incorporates the uncertainty values of the input features and class labels to reduce the cumulative class heterogeneity in the resulting child nodes.

For making predictions on new data, the data points are propagated through the trees and reach all the terminal nodes with some probability. The final class prediction is calculated as the average of the class probabilities generated by all the terminal nodes.

### 2.6. Model training and hyperparameter selection

We used a nested and stratified 7-fold cross-validation (CV) scheme for hyperparameter selection, model training, and evaluation of the trained model for generalizability. In nested CV, the outer loop divides the data into training and testing splits for model evaluation while the inner loop further divides the training split into training and validation splits for hyperparameter selection. Stratification maintains roughly the same class frequencies across all folds. In each outer loop, data from 6-fold (*n* = 122) was used to train the classifier while the remaining data from the 7-fold (*n* = 20) was used to evaluate the model using performance metrics described in the following section. In each inner loop, we used a 7-fold CV with stratification again to select the hyperparameters for optimizing the model’s performance, using 6-fold (*n* = 104) for training and one validation fold (*n* = 18) to evaluate the model’s performance. The hyperparameters selected using CV included the number of trees, the number of features for node splitting, the minimum sample size for node splitting, and the minimum sample size to be maintained in leaf nodes. The last two hyperparameters do not apply to the PRF as the uncertainty distribution and another hyperparameter *keep_probability* dictate whether a data point is propagated to either both child nodes or a single node. We used the default value for *keep_probability* (0.05).

The combination of hyperparameters that produced the best average performance over the seven validation folds was then selected as the final model for the given outer fold and was tested for generalizability using the held-out test fold. The reported performance metrics were calculated by averaging the model’s performance over the seven test folds.

It should be noted that the prevalence of individuals who developed new disease activity within 24 months in the confirmed label subgroup was 74.07%. To address class imbalance during model training, we used class weights, informed by class frequencies in the training sample, for calculating the loss function. This allowed the model to learn from both the majority and minority classes in the training sample in a balanced way.

### 2.7. Implementation details

The experiments and data analyses were performed using Python 3.7.5. Data processing was performed using Pandas 0.25.3 and NumPy 1.14.5. The RF classifier was built and trained using Scikit-learn 0.23.1. The PRF classifier was built and trained using the PRF library (44).

### 2.8. Class probabilities for PRF

As previously discussed, a PRF takes as input the probability of a sample belonging to the given class. For example, in our dataset, a target probability $\Delta y_{i}=0.3$ for a data sample that had a *post hoc* class label would indicate that the subject had a 30% probability of belonging to the stable class and a 70% probability of belonging to the new-disease-activity class. Ideally, every data sample with an uncertain label would have a probability estimated from prior knowledge (e.g., population studies); however, in this case, we did not have sufficient background information to calculate the probabilities, so we assigned a 50% probability to all of the *post hoc* labels. For the samples with confirmed labels, we assigned a 100% class probability.

### 2.9. Evaluation of model performance and statistical analysis

To provide a reliable estimate of model performance, we have reported the evaluation metrics on test data with confirmed labels only. The overall performance of a classifier on any metric was calculated by averaging its performance over the seven test folds. Since our dataset was unbalanced, the primary measures of model performance were the F1-score and the area under the receiver operating curve (ROC). The F1-score is a harmonic mean of precision and recall (sensitivity) and gives a more holistic estimate of a model’s performance on prediction over both classes and is therefore a more robust indicator of performance compared to accuracy. F1-score was calculated by the following formula:


$$F1-score=\frac{2*\left(\mathrm{Precision}*\mathrm{Recall}\right)}{\left(\mathrm{Precision}+\mathrm{Recall}\right)}$$

where


$$Precision=\frac{TP}{\left(\mathrm{TP}+\mathrm{FP}\right)}$$

$$Sensitivity=\frac{TP}{\left(\mathrm{TP}+\mathrm{FN}\right)}$$

TP, FP, and FN denote True Positive, False Positive, and False Negative, respectively.

A ROC is a plot between a classifier’s true positive rate (TPR) and False Positive Rate (FPR) and gives a visual depiction of the trade-off between a model’s ability to correctly identify both classes. The area under the ROC (AUC) summarizes the classifier’s overall ability to separate the negative and positive classes correctly. The TPR and FPR are calculated using the following formulae.


$$TPR=\frac{TP}{\left(\mathrm{TP}+\mathrm{FN}\right)}$$

FNR=1−TPR

## 3. Results

The performance metrics for the three classifiers (RF with confirmed labels only, RF with all samples and assuming *post hoc* labels were correct, and PRF with all samples) are summarized in Table 3.

**Table 3:** Comparison of mean (SD) performance metrics averaged over stratified 7-fold cross-validation.

Approach	AUC	Recall	Precision	F1-Score
RF_exclude_ (trained with only the 110 confirmed labels)	0.69 (0.09)	90.15 (5.26)	77.02 (4.95)	82.58 (5.94)
RF_naive_ (trained with all 142 samples, with 32 uncertain labels assigned as stable)	0.71 (0.16)	71.97 (8.98)	**86.42 (5.17)**	76.82 (6.57)
PRF (trained with all 142 samples, with 32 uncertain labels assigned a 50% probability)	**0.76 (0.14)**	**92.32 (4.22)**	82.31 (7.78)	**86.57 (6.71)**

In each fold, the model performance was measured on a test set with only confirmed labels. Bold indicates the highest values.

**Figure 1:**
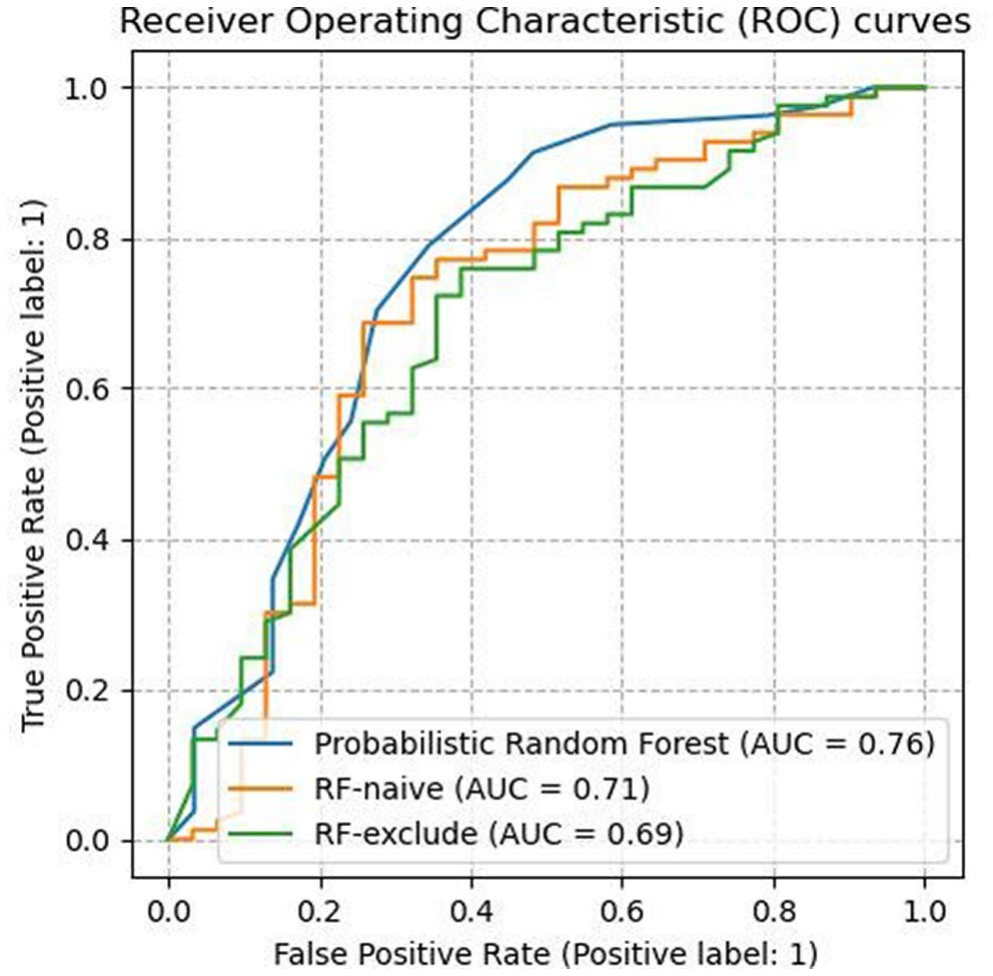
The ROC curves for all three classifiers. PRF clearly outperformed RF_exclude_ and RF_naive_.

As shown in Table 3, the PRF trained using the entire dataset outperformed both of the other RF classifiers across all evaluated metrics except precision. It yielded an AUC score of 0.76 (SD = 0.14) which was an improvement of 0.07 compared to 0.69 (SD = 0.09) achieved by RF_exclude_ and 0.05 compared to 0.71 (SD = 0.16) by RF_naive_. Figure 1 shows the ROC curves for all three classifiers, with a clear advantage shown by PRF. PRF also achieved the highest F-1 score of 86.57% (SD = 6.71%), which was an improvement of 3.99% compared to 82.58% (SD = 5.94%) by RF_exclude_ and 9.75% compared to 76.82% (SD = 6.57) by RF_naive_. Overall, RF_naive_ performed worst compared to the other two classifiers, except for precision where it achieved the highest score of 86.42% (SD = 5.17) vs. 82.31% (SD = 7.78) by PRF and 77.02% (SD = 4.95) by RF_exclude_. RF_exclude_ and RF_naive_ did not differ in AUC, but all differed across all other measures.

Accounting for label uncertainty by either excluding the uncertain samples or modeling the uncertainty resulted in a marked improvement in the classifier’s sensitivity. PRF achieved a recall score of 92.32% (SD = 4.22%) and RF_exclude_ achieved 90.15% (SD = 5.26%), resulting in improvements of 20.35 and 18.18% respectively, over the 71.97% (SD = 8.98) produced by RF_naive_. The observation that RF_naive_ had the highest precision but lowest recall demonstrates a classic trade-off between precision and recall, but accounting for label uncertainty increased recall dramatically for PRF and RF_exclude_, while decreasing precision to a lesser degree, especially in the case of PRF.

In terms of feature importance, the burden of disease and the volume of thalamic nuclei were consistently ranked as two of the most informative input features by all the models used in the study. This finding is in line with existing literature that has identified thalamic nuclei and white matter lesion load as important predictors of disease progression in early MS. (9, 10, 42)

## 4. Discussion

We have evaluated and compared three approaches to account for uncertainty in missing class labels when using ML to predict MS disease activity: (i) exclusion of data samples with uncertain labels; (ii) replacing uncertain labels with assumed labels; and (iii) using a classifier with an inherent capability to model label uncertainty (PRF). Exclusion is the most common approach for dealing with uncertain data points, but this may not be an optimal approach for medical imaging datasets, which tend to be small for ML purposes. Eliminating data points from an already small dataset increases the risk that the ML algorithm cannot learn the distribution of the samples. Therefore, there is practical value in investigating methods that make use of all available data points while modeling any uncertainty in the class labels.

Our experiments have shown that the RF classifier naively trained on a dataset comprising of both confirmed and uncertain class labels (RF_naive_) performed the worst overall. It achieved the lowest F1-score indicating that it was not able to learn an effective decision boundary between true and false positives, in this case, favoring precision while greatly sacrificing sensitivity, which is detrimental to early disease detection. The results demonstrate that the input features of data points with uncertain labels can be informative and help the model learn distinctive patterns that produce better decision boundaries.

Probabilistic random forest has various advantages over RF. PRF can intrinsically handle missing values, both for input features as well as for class labels. Data points with missing input features are propagated to both the child nodes after a node split. In the case of missing class labels, the class probabilities, along with the hyperparameter *keep_probability*, dictate whether a data point is propagated to both child nodes and a single node. Clinical datasets are generally small for machine learning applications and therefore a machine-learning model that can handle missing values without reducing the size of the dataset is potentially highly beneficial since missing variables are common in data from electronic health records (EHRs) and clinical studies.

To our knowledge, only two other studies have compared the performance of PRF with other classifiers in a practical application. One study by Mervin et al. (33) trained an RF and PRF to predict protein-ligand interaction using bioactivity data. For training the PRF, they incorporated the uncertainty associated with the class labels by using the standard deviations in the experimental measures used to assign the labels to calculate the class probability values. They showed that the PRF outperformed the traditional RF and was more tolerant of the levels of uncertainty in the class labels. Another study using photometric astronomy data (45) showed that in the presence of missing input features, PRF outperformed canonical correlation analysis for classification.

One key challenge with training a PRF on a dataset with missing labels is that one needs to specify the class probabilities. For medical data, where the outcome of interest is a diagnosis, phenotype, or other clinical outcomes, the class probabilities could potentially be informed by the statistical prevalence of the outcomes reported in the literature, if the dataset could be assumed to be representative of the study population at large. In the absence of such information, assigning a 50–50 class probability may be a reasonable choice, as we have done in our study.

The focus of our study was on CIS and early MS, but the methods employed are potentially applicable to RIS. Individuals with RIS have MRI findings (white matter lesions demonstrating dissemination in space) that are consistent with MS disease in the absence of clinical symptoms of MS. (46) Despite some recent large studies that have shed light on several key risk factors for conversion to MS (47, 48), prediction on an individual level remains difficult. Previous studies (11, 49) investigating DGM volumetric changes in RIS have shown that thalamic volumes were significantly lower in individuals with RIS than in normal controls. Applying ML to DGM changes can potentially discover patterns with other DGM structures, possibly in combination, that can help with the prediction of conversion of RIS to MS or for risk stratification for early management. Accounting for uncertainty in RIS would be important because sample sizes of RIS studies tend to be small for ML purposes.

## 5. Limitations to the study

Our study has several limitations. Even though we maximized the use of our data by employing stratified CV, the sample size of our dataset is such that the results only serve as a proof of concept for our hypotheses and further investigations with larger datasets are needed to further validate the generalizability of the findings. Another limitation is that we did not explore the ability of PRF to model noise in the input features. Experiments by (32, 33) have shown that PRF is more robust to noise than RF across various degrees of noise. While we have focused only on uncertain outcome labels in this study, we intend to explore the potential benefit of PRF for noisy input features in the future.

## 6. Conclusion

In the context of predicting disease activity in CIS/early MS using machine learning, we have demonstrated that in a dataset with uncertain outcomes labels, using a method designed to model the uncertainties can produce substantially improved performance over the common approach of excluding the uncertain samples.

## Data availability statement

The data analyzed in this study are subject to the following licenses/restrictions: data were collected as part of a clinical trial without an open-access policy. Requests to access these datasets should be directed to RT (roger.tam@ubc.ca).

## Ethics statement

The studies involving human participants were reviewed and approved by UBC Clinical Research Ethics Board. The patients/participants provided their written informed consent to participate in this study.

## Author contributions

MT was responsible for developing the analysis pipeline, implementing the machine learning models, performing all the experiments, and drafting the manuscript. LM was the principal investigator of the minocycline trial (38) and provided all clinical information on the participant groups. DL, SK, AT, and RT are members of the multiple sclerosis/magnetic resonance imaging (MSMRI) research group at the University of British Columbia, which was the core image analysis laboratory for the minocycline trial and performed the MRI volumetric measurements. LM, DL, SK, RC, and AT provided substantial input on clinical motivation, experimental design, interpretation of results, and manuscript revisions. RT provided primary supervision of all aspects of this study. All authors contributed to the article and approved the submitted version.

## Funding

MT and RT were supported by an NSERC Discovery grant (RGPIN-2018-04651).

## Conflict of interest

The authors declare that the research was conducted in the absence of any commercial or financial relationships that could be construed as a potential conflict of interest.

## Publisher’s note

All claims expressed in this article are solely those of the authors and do not necessarily represent those of their affiliated organizations, or those of the publisher, the editors and the reviewers. Any product that may be evaluated in this article, or claim that may be made by its manufacturer, is not guaranteed or endorsed by the publisher.
